# Author Correction: Late Cambrian geomagnetic instability after the onset of inner core nucleation

**DOI:** 10.1038/s41467-023-40882-x

**Published:** 2023-09-04

**Authors:** Yong-Xiang Li, John A. Tarduno, Wenjun Jiao, Xinyu Liu, Shanchi Peng, Shihua Xu, Aihua Yang, Zhenyu Yang

**Affiliations:** 1 State Key Laboratory for Mineral Deposits Research, Institute of Continental Geodynamics, School of Earth Sciences and Engineering, Nanjing University, Nanjing, 210023 China; 2 Department of Earth & Environmental Sciences, University of Rochester, Rochester, NY USA; 3 Department of Physics & Astronomy, University of Rochester, Rochester, NY USA; 4 Laboratory for Laser Energetics, University of Rochester, Rochester, NY USA; 5 State Key Laboratory of Geology and Palaeontology, Nanjing Institute of Palaeontology, Chinese Academy of Sciences, Nanjing, 210008 China; 6 College of Resources, Environment & Tourism, Capital Normal University, Beijing, 100048 China

**Keywords:** Palaeomagnetism, Core processes, Palaeontology, Geomagnetism, Stratigraphy

Correction to: *Nature Communications* 10.1038/s41467-023-40309-7, published online 31 July 2023

The original version of this Article contained an error in Figure panel 1e, which incorrectly defined ‘l’ as ‘l = 44.2°’ The correct version states ‘l = −44.2°’. This has been corrected in both the PDF and HTML versions of the Article.

