# Supplementary material for: Late Cambrian geomagnetic instability after the onset of inner core nucleation
Source: Nat Commun. 2023 Jul 31;14:4596. doi: 10.1038/s41467-023-40309-7 (PMC10390560; doi:10.1038/s41467-023-40309-7)
Supplement: Supplementary file 1 — Supplementary Information [file 41467_2023_40309_MOESM1_ESM.pdf]

Supplementary Information (SI) *of*

Late Cambrian geomagnetic instability after the onset of inner core nucleation

Yong-Xiang Li[1](yxli@nju.edu.cn), John Tarduno[2,3,4], Wenjun Jiao[1], Xinyu Liu[1], Shanchi Peng[5], Shihua Xu[1], Aihua Yang[1], Zhenyu Yang[6]

1. State Key Laboratory for Mineral Deposits Research, Institute of Continental Geodynamics, School of Earth Sciences and Engineering, Nanjing University, Nanjing 210023, China
2. Department of Earth & Environmental Sciences, University of Rochester, Rochester, NY, USA.
3. Department of Physics & Astronomy, University of Rochester, Rochester, NY, USA
4. Laboratory for Laser Energetics, University of Rochester, Rochester, NY, USA
5. State Key Laboratory of Geology and Palaeontology, Nanjing Institute of Palaeontology, Chinese Academia of Science, Nanjing 210008, China
6. College of Resources, Environment & Tourism, Capital Normal University, Beijing 100048, China

This Supplementary Information (SI) contains four sections of supplementary text and nine supplementary figures. The four sections are: 1. Prior interpretations for the onset of inner core nucleation (ICN); 2. Geological setting; 3. Sedimentation rate of the studied interval; 4. Results. Supplementary figures 1-9 are embedded in the supplementary text.

1. Prior interpretations for the onset of inner core nucleation (ICN)

Here we briefly review other relevant modeling and data-driven interpretations on the age of ICN published since 2016. Landeau et al.[1] offered models with ICN at approximately 700 Ma, which showed no observable surface intensity change upon ICN, but instead a long-term decrease in field strength from the time of ICN to the present-day. This ICN age is compatible with our interpretations on field instability, core size, and TPW artifacts. But the predicted intensity history is incompatible with paleointensity data[2,3] and therefore should not be considered a viable description of Earth's Precambrian to recent dynamo.

In the evaluation of ICN ages based on observations, fundamental requirements of paleointensity data must be considered. First, because of the temporal and spatial variations of the geomagnetic field, time-averaging is needed to learn about the geodynamo[cf. 2,3]. Specifically, values from lavas or dikes represent only instantaneous snapshots of the geomagnetic field, and thus many time-independent values spanning some tens-of-thousands of years are needed. Alternatively, slowly cooled rocks can provide inherently time-averaged data. Second, single-domain like magnetic grains are required in all samples analyzed because thermoremanent magnetization theory, needed to ground paleointensity interpretations, is available only for these magnetic carriers[4]. Third, Thellier double heating experiments are needed to reliably recover the field[4].

(1) Ediacaran ICN

Bono et al.[2] linked the Ediacaran to ICN based on the extremely low paleointensity value and coeval unusual directional behavior in Ediacaran. In particular, Bono et al.[2] noted a 2 billion-year-long paleointensity decrease leading into the Ediacaran Period. This is important and lends further support to the Ediacaran as the time of ICN, because the thermally driven dynamo is expected to wane with time. Specifically, Bono et al.[2] stated "With the caveats that only a few time-averaged values are

available and bulk rocks contain non-ideal magnetic carriers, a trend to a lower field strength from Archaean to Ediacaran times, with superimposed variability, is hinted at by a new database of reliable Precambrian palaeointensity values". The database referred to was provided by Bono et al.[2] and the trend calculated was weighted to time-averaged values.

(2) ~1.1 Ga ICN or Devonian ICN

Zhang et al.[5] suggested an ICN age of 1.1 Ga by comparison of data from the Mid-Continental rift and results from the Abitibi dikes[6]. Again, this ICN age is compatible with our interpretations. However, the Abitibi dike data are not time-averaged, and rock magnetic concerns have been raised; specifically results on similar dikes suggest the magnetic recorders could yield data biased to low values[7]. In addition, as noted by Zhang et al. [5], the nominal Abitibi results do not define an ultralow field. Therefore, even if the Abitibi results are confirmed with robust magnetic recorders, we view this as within the range of a highly variable geomagnetic field, and a signal of ICN. Zhang et al.[5] interpreted low paleointensity values reported for the Devonian Period[8] as a signature of ICN. We note these data also are not time-averaged, limited Thellier data are available, and while some instantaneous values are low (but not ultralow) others are higher. Furthermore, all paleointensity reports on the Devonian rocks comment on the difficulty in obtaining reliable values due to common remagnetizations. We agree with interpretation of the available Thellier values recording low fields as fluctuations of dynamo efficiency related to core-mantle boundary processes, as interpreted for Mesozoic to recent times[8-10].

Zhang et al.[5] criticized the Bono et al.[2] 2 billion-year-long paleointensity trend based on their new results from the Mid-Continent rift stating "The high paleointensity estimates from the 1.1-Gy-old Mid-continent Rift rocks challenge the hypothesized monotonic decay of the geomagnetic field strength throughout the Proterozoic Era (Fig. 6)…Overall, the anorthosite xenoliths from this study record a high Mesoproterozoic field exceeding the value projected by the second-order polynomial curve from ref. 22 which is based on an interpretation of there being a monotonic decay of the geodynamo through

the Proterozoic." But, Zhang et al. erred in two important ways. First, they compared their new instantaneous values with a fit calculated by Bono et al.[2] and weighted to time-averaged values; when the new data of Zhang et al.[5] are combined with the much larger prior Mid-Continental Rift paleointensity data set of Kulakov and Smirnov[11], the resulting mean value cannot be distinguished from that used by Bono et al.[2], and therefore the trend is unchanged. Second, Bono et al.[7] did not interpret the trend as "monotonic", but recognized variations, as these are expected, reflecting changes in core-mantle boundary conditions as discussed above. The Mid-Continental rift data are simply one such variation. Zhang et al.[5] also highlight results from the ca. 755 Ma Mundine Well dikes, claiming that these "…also require a stronger geomagnetic field in the Neoproterozoic than would be predicted by a progressive Proterozoic decline". However, this result does not contain sufficient Thellier data to meet selection criteria[2,3]. Therefore, when data are properly scrutinized, and time-averaged, the Bono et al.[2] trend remains as a viable first-order description of the available reliable time-averaged data, and this trend further supports an Ediacaran ICN age.

(3) Cambrian or younger ICN?

Recently, Lloyd et al.[12] reported a nominal low paleointensity value at 532 Ma. When compared to the much higher time-averaged value reported by Zhou et al.[3], this could reflect continued field instability in the early Cambrian, as we invoke for the late Cambrian. However, we urge caution over this result because the authors report that the rocks studied were hit by lightning. Since lightning strike fields can exceed the coercivities of all terrestrial magnetic minerals[4], rocks hit by lightning are usually excluded from paleointensity investigations.

2. Geological setting

The studied Cambrian succession is located in western Duibian village (N28º48.958', E118º36.896'), Jiangshan County, Zhejiang Province in South China (Fig. S1). Tectonically, South China is composed of the Yangtze craton to the northwest and the Cathysia block to the southeast. The two blocks amalgamated

in the Neoproterozoic and became one tectonic entity by ~820 Ma[13-15]. Rifting took place during ~800 Ma to ~750 Ma, leading to extensive deposition in northeasterly elongated rift basins[16,17]. Rifting was followed by tectonic quiescence until the late Ordovician when the Kwangsian Movement occurred[18], which deformed and uplifted Neoproterozoic to early Paleozoic strata, resulting in a regional angular unconformity. The subsequent Indosinian and Yanshanian orogenies further deformed Paleozoic strata in South China[17].

Paleogeographically, the Cambrian marine succession studied accumulated on the outermost part of the NE-trending Jiangnan Slope[19] and probably near the Zhe-Min Land to its southeast[20]. The Jiangnan Slope demarcates deposits on the Yangtze carbonate platform to the northwest from the hemipelagic deposits of the adjacent Jiangnan Basin to the southeast[19,21]. The section investigated is situated at the southeastern limb of a syncline with a NE striking fold axis (Fig. S1). The section consists of the Hotang, Dachenling, Yangliugang, Huayansi, and Siyangshan formations (Fm)[22]. The Global boundary Stratotype Section and Point (GSSP) for the base of Cambrian Stage 9 lies in the section studied, coinciding with the first appearance datum (FAD) of agnostoid trilobite *Agnostotes orientails*[22]. The stage defined by the GSSP was ratified as the global Jiangshanian Stage, the second stage of the Furongnian Series, Cambrian System, by the International Union of Geological Science (IUGS) in 2011[23]. The Jiangshanian GSSP occurs in the Huayansi Fm that is composed of marine carbonate rocks. The Cambrian strata in South China experienced episodes of deformation mainly during the Caledonian and Indosinian orogeny[24,25]. In spite of the deformation, the Cambrian strata of the study area did not undergo metamorphism or strong diagenetic alteration[23,24].

3. Sedimentation rate of the studied interval

The sedimentation rate was calculated based on[26] and the ages of Jiangshanian Stage and Paibian Stage in the Geological Time Scale (GTS). There are two sections, Duibian A section and Duibian B section, in the study area (Fig. S1). The Jiangshanian GSSP is from the Duibian B section and can be traced to the Duibian A section[26] (Fig. S1). Our studied section is from the Duibian B section (Fig. S1). Our studied interval is mainly in the upper part of the Paibian Stage (Fig. 1, Fig. S1). Therefore, the sedimentation rate

is calculated for the Paibian Stage in the study area. The thickness of the Paibian Stage in the Duibian A section is ~96 m (Fig. 5 of ref.[26]). According to the GTS2012 for the Cambrian period[23], the age for the upper bound of the Paibian Stage, i.e., the Jiangshanian GSSP, is ~494 Ma, and the age for the lower bound of the Paibian Stage is ~497 Ma. The mean sedimentation rate is then estimated to be ~3.2 cm/kyr. The GTS2020 shows the ages of the GSSPs of Jiangshanian and Paibian Stages are ~494.2 Ma and ~497 Ma, respectively[27], indicating a duration of ~2.8 Myr for the Paibian Stage and leading to an estimation of ~3.4 cm/kyr for the Paibian Stage in the study area. More recently, it is shown that Paibian Stage may contain 7.5 to 8 long eccentricity cycles (~405 kyr), indicating a duration of ~3.2 Myr (Fig. 2 and Fig. 4 of ref.[28]), which would lead to an estimate of ~3.0 cm/kyr for the study area. So the estimated sedimentation rates could be ~3.2 cm/kyr, ~3.4 cm/kyr, or ~3.0 cm/kyr using different geological timescales. Given the age uncertainties of each timescale used, we chose the medium value, ~3.2 cm/kyr, as the sedimentation rate for the study interval to discuss our results in this study.

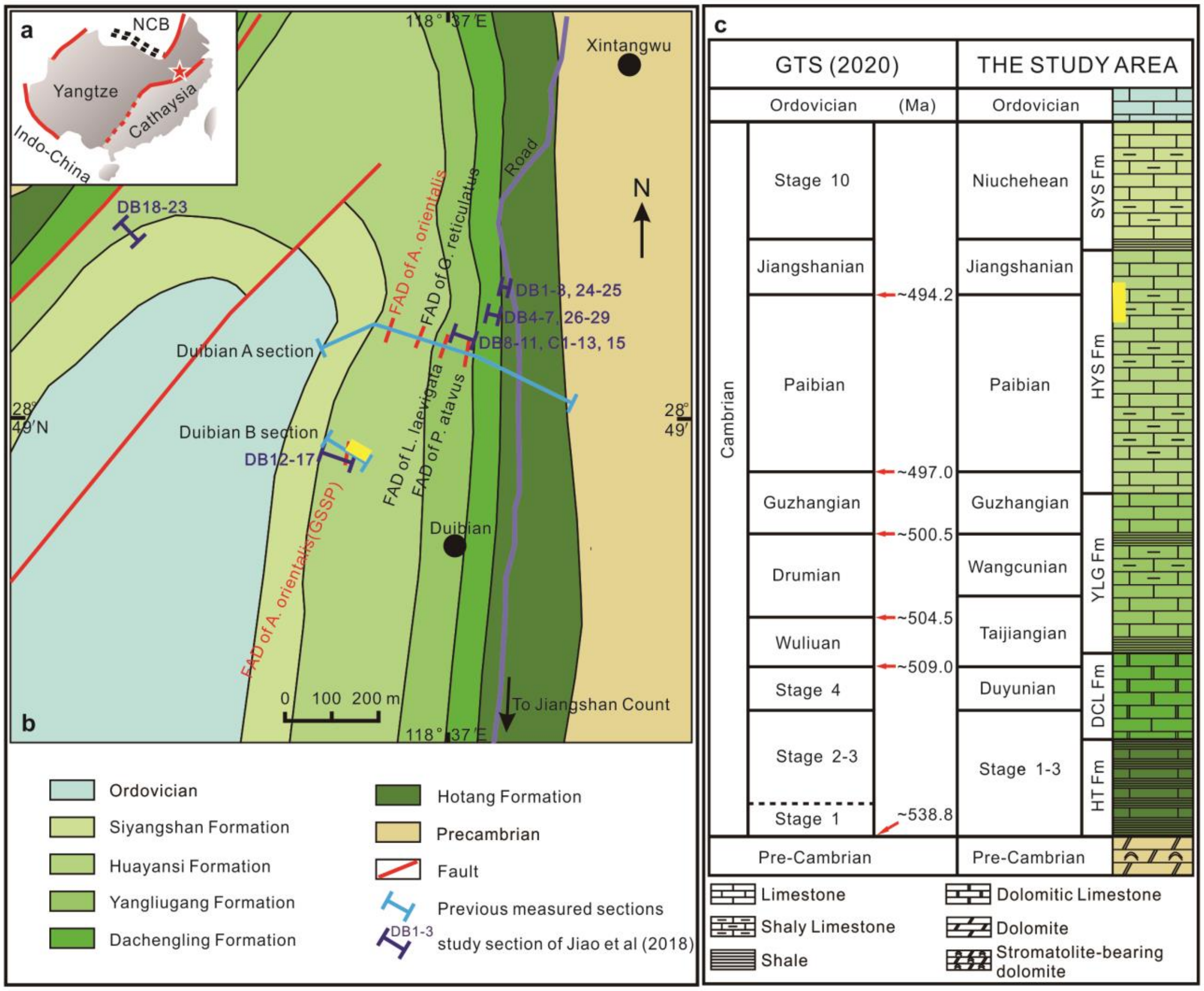

Figure S1. Map shows the location and the stratigraphy of the Jiangshanian GSSP section in Jiangshan, South China. The yellow bars mark the chronostratigraphic interval of the high-resolution magnetostratigraphic investigation of this study. HT Fm, Hotang Formation; DCL Fm, Dachengling Formation; YLG Fm, Yangliugang Formation; HYS Fm, Huayansi Formation; SYS Fm, Siyangshan Formation. Figure modified after Jiao et al.[35]. GTS2020, Geological Time Scale 2020 for the Cambrian Period after Peng et al.[27].

4. Results

4.1 Rock magnetic data

Thermal demagnetization has been shown to be more effective than alternating field (AF) demagnetization in isolating remanence components of the studied section[35] (Fig. S2). Thermal demagnetization results show that the majority of remanence is removed by 500°C. Thermal demagnetization of the composite IRM shows that the remanence is dominantly carried by minerals with coercivities less than 0.125 T (Fig. S3). In addition, the decay of the 0.5 T component between 300-350°C is rather gradual and not typical of the sharp change typically seen in pyrrhotite[4]. Together, these data suggest that magnetite grains of different sizes are likely the major remanence carriers. But as described below, Al and Cr substitution may be common in the grains, lowering unblocking temperatures. Also, we note that occasionally the samples show evidence for a higher coercivity phase that is not fully demagnetized at temperatures of ~625°C, which we interpret as hematite (Fig. S3b). Magnetic hysteresis loops of selected samples from the studied interval show that the majority of samples can be magnetically nearly saturated by ~100 mT (Fig. S4). Two samples from 8.7 m and 7.87 m show hysteresis loops with a saturation field of > ~300 or 400 mT (Fig. S4), suggesting the presence of magnetic minerals of high coercivity, which again may point to a contribution of hematite. This is arguably more pronounced by the wasp-waisted nature of the curves[29] which can be the result of mixtures of magnetite and fine-grained hematite in limestones[30,31]. On a Day plot[32-34], the magnetic hysteresis data of all samples fall in the pseudosingle domain (PSD) field, suggesting that the dominant grains are in PSD domain state or a mixture of single domain (SD) and multidomain (MD) states. Together with the wasp-waistedness of hysteresis loops, grains with contrast sizes are likely present.

Most rock magnetic parameters measured for the interval between 0 m and ~9.5 m (see Methods) indicative of magnetic mineralogy, concentration, and grain size show only subtle variations around their mean values (Fig. 2a-e) (Table S2). An exception is the S-ratio, which shows relatively large variations above 7.3 m, but these are within Interval IV of stable normal polarity and not germane to the variable field behavior defined lower in the section. Although not a precise relative paleointensity estimate, the

NRM/ARM and NRM/SIRM ratios of the rock magnetic samples (Fig. 2f, 2g) are sufficient to detect whether there are intervals of anomalous intensity for the following two reasons. First, NRM, the numerator, is proportional to NRM350°C, around which the start of ChRM is chosen, of the thermally demagnetizated paleomagnetic samples except at ~8.0 m where there is a spike (Figs. S6a and S6b). Second, ARM and IRM, the normalizers, of the rock magnetic samples exhibit strong correlation (Fig. S6c). The spike at ~8.0 m might be mineralogical or intensity anomalies, but it is within Interval IV and does not affect the conclusions about Interval III.

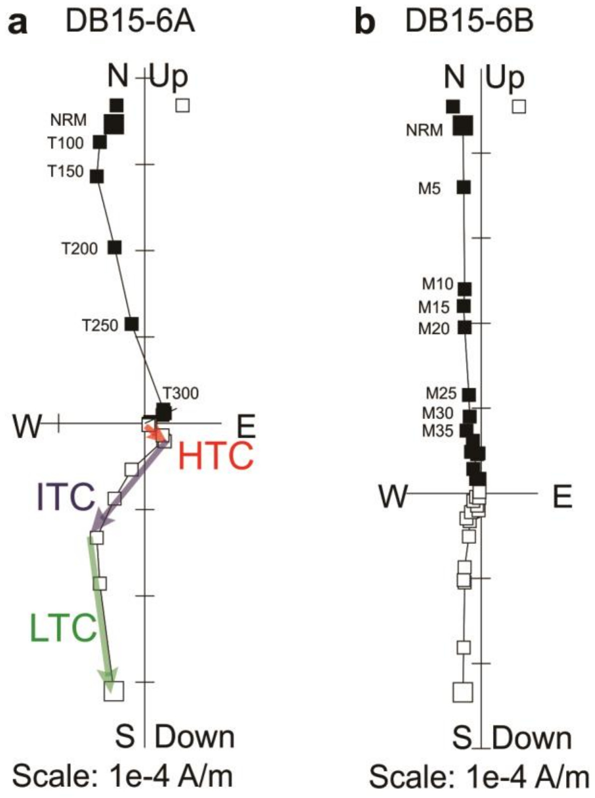

Figure S2. The demagnetization data of two sister specimens from ~19 m of the studied section showing that the HTC can be well resolved by thermal demagnetization (**a**), but cannot be resolved by AF demagnetization (**b**). Therefore, thermal demagnetization is more effective in isolating HTC than AF demagnetization.

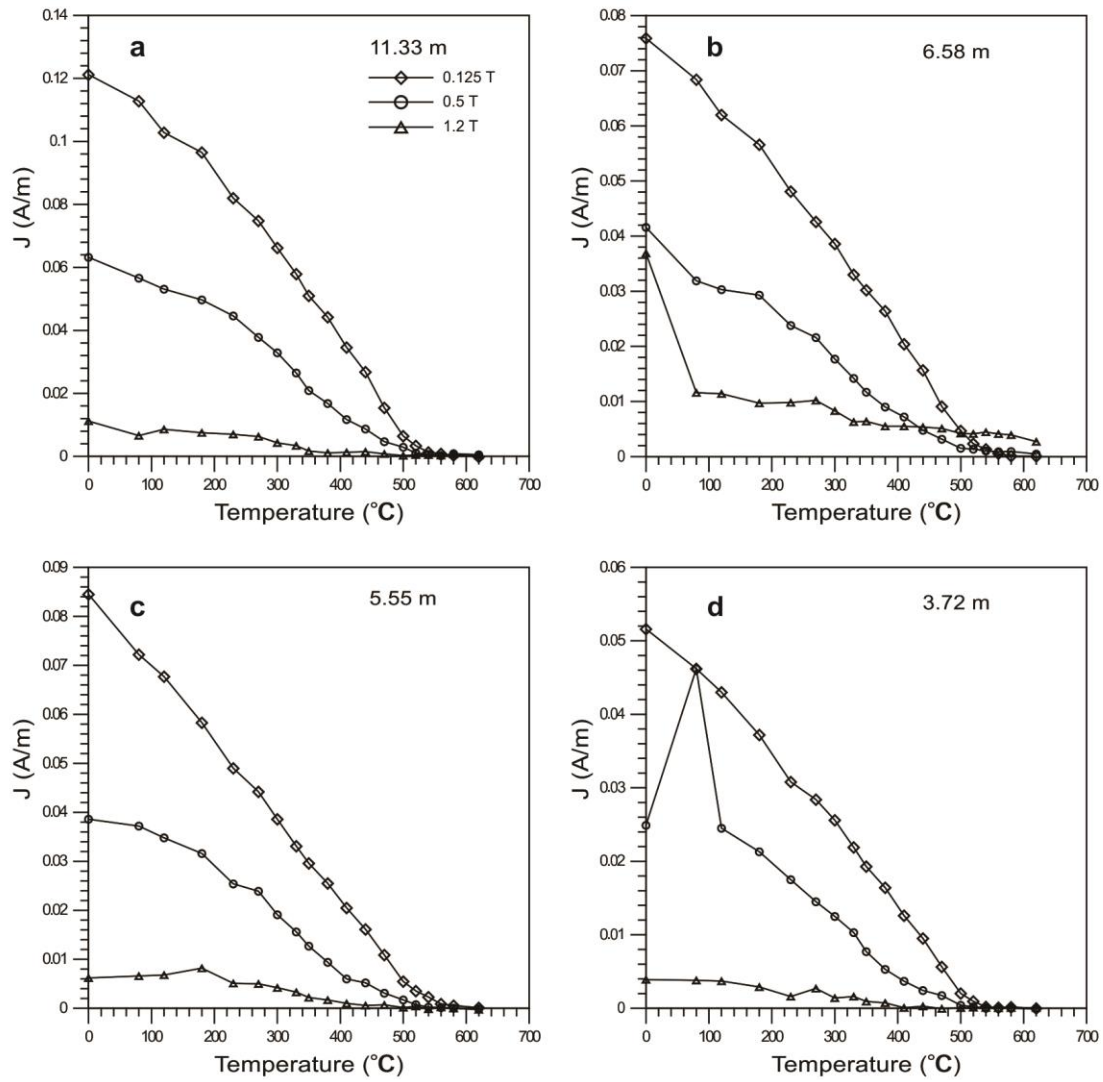

Figure S3. Stepwise thermal demagnetization results of the composite isothermal remanent magnetization (IRM) acquired successively along z-, y-, and x-axis. The samples from the normal (**a**), transitional (**b, c**), and reversed (**d**) polarity intervals show similar features.

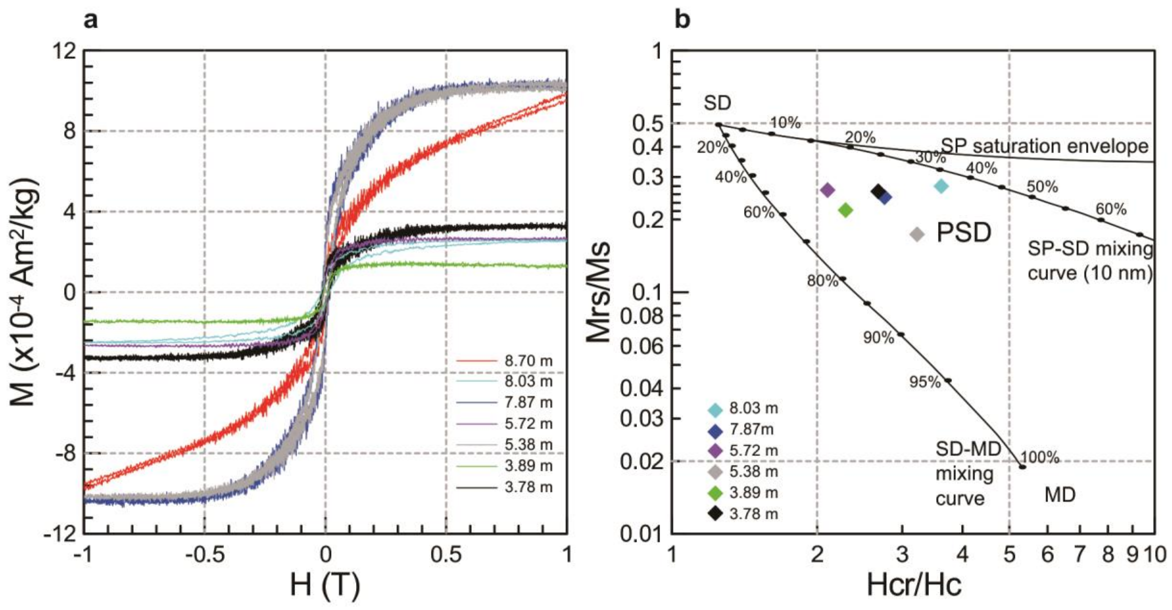

Figure S4. The magnetic hysteresis loop data and Day plot[32-34] of selected samples of the investigated interval in the Jiangshanian GSSP section, South China.

4.2 Scanning Electron Microscopic (SEM) observations

Jiao et al. (2018)[35] reported TEM data from the upper Cambrian strata (Fig. 8 of Jiao et al., 2018[35]) that show the occurrence of magnetite grains. To further examine the magnetic mineralogy of the studied section, we have carried out a systematic investigation of scanning electron microscopic (SEM) observations along with energy dispersive spectroscopy (EDS) analyses of 2, 3, 6, 3 selected samples from Intervals I, II, III, and IV, respectively. The common features of the SEM data are summarized as follows. There are magnetite grains in all of the four intervals. These grains occur in voids in the carbonate and are subangular to subrounded in shape, suggesting a detrital origin (Fig. S5 Part 1). The occurrence of Al or Cr peaks is common, suggesting substitution of these elements into the magnetite lattice (Fig. S5 Part 2). Pyrite (FeS_2) grains also occur in all the four intervals and these grains appear either in euhedral crystals or framboids of small equal-sized particles (Fig. S5 Part 3). The common occurrence of these pyrite grains is

consistent with anoxic conditions during deposition or shortly thereafter.

More detailed SEM observations were conducted for Interval III because this interval documents an ~90° directional shift. Five samples (4.87 m, 5.26 m, 5.52 m, 5.78 m, 6.93 m) that recorded the directional shift and one sample (5.96 m) that yields an outlier direction were examined. The five samples recording the direction shift show magnetite grains of subangular to subrounded shape in voids of carbonate (Fig. S5 Part 1), indicative of a detrital origin of these grains. A few grains also contain Ti, indicating titanomagnetite (Fig. S5 Part 5c). In addition to ubiquitous pyrite, rutile (TiO_2) is also common in these samples (Fig. S5 Part 4). Like the magnetite grains, the rutile grains appear detrital based on their morphology. As noted above, pyrite grains are present as either euhedral crystals or framboids. Thus, the SEM-EDS characteristics of these five samples from Interval III are similar to those of the other intervals.

In contrast to these five samples from Interval III, the sample recording an outlier direction at 5.96 m shows a different magnetite occurrence. One sub-euhedral crystal displays what appears to be a small irregular patch containing Fe and S in an otherwise Fe oxide grain (Fig. S5 Part 5a). This could record partial alteration of pyrite resulting in magnetite; however, other interpretations are possible, including processes prior to deposition. A clearer example of transformation of pyrite to magnetite, however, is observed in a framboidal occurrence (Fig. S5 Part 5b). We suggest that the alteration of pyrite to magnetite caused this sample to acquire a secondary remanence whose direction occurs as an outlier among the data that show a directional shift in Interval III. The process causing this transformation would likely have been the introduction of oxidizing fluids.

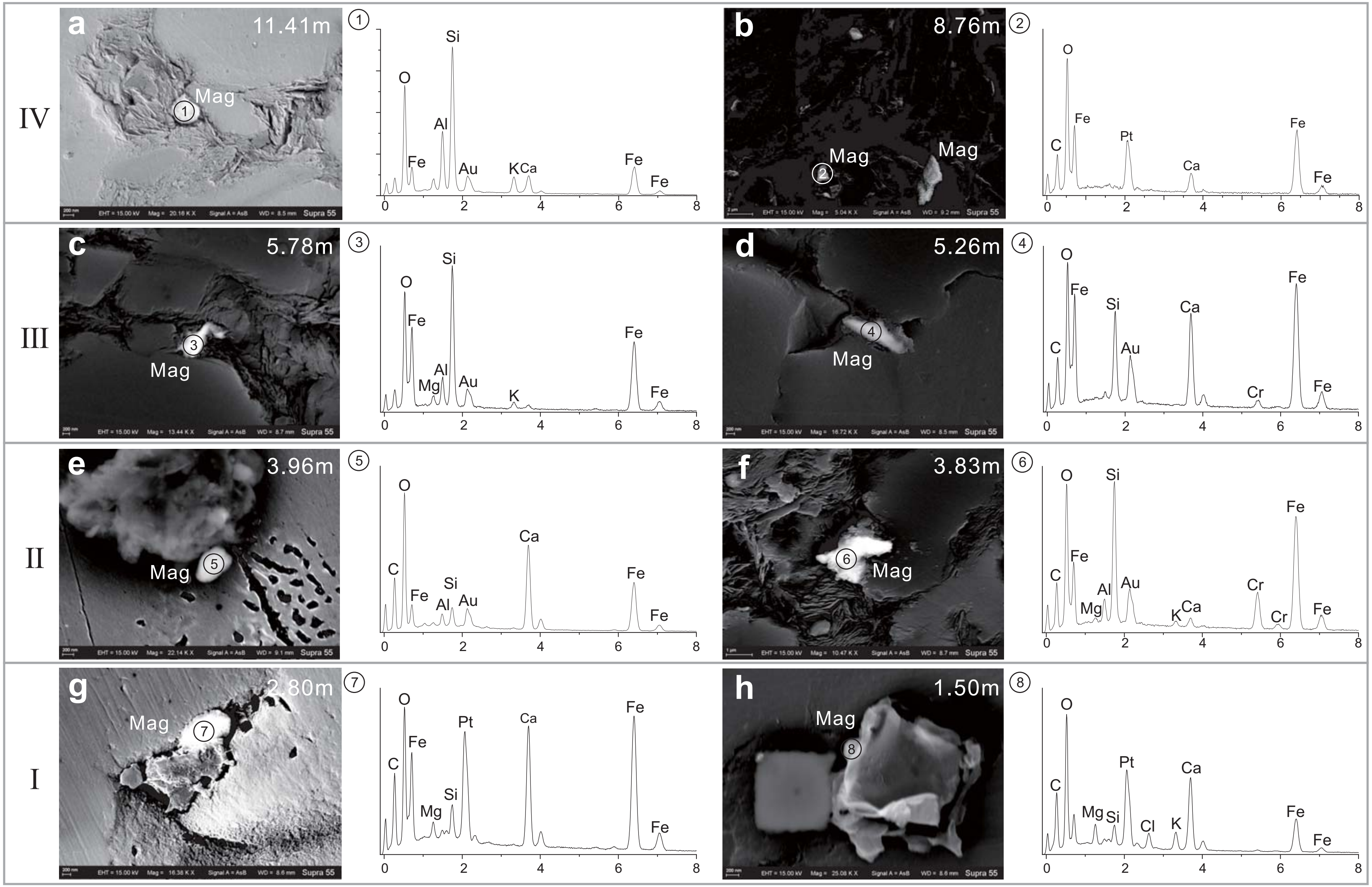

Figure S5 Part 1. SEM images of samples from the four intervals showing the occurrence of detrital magnetite

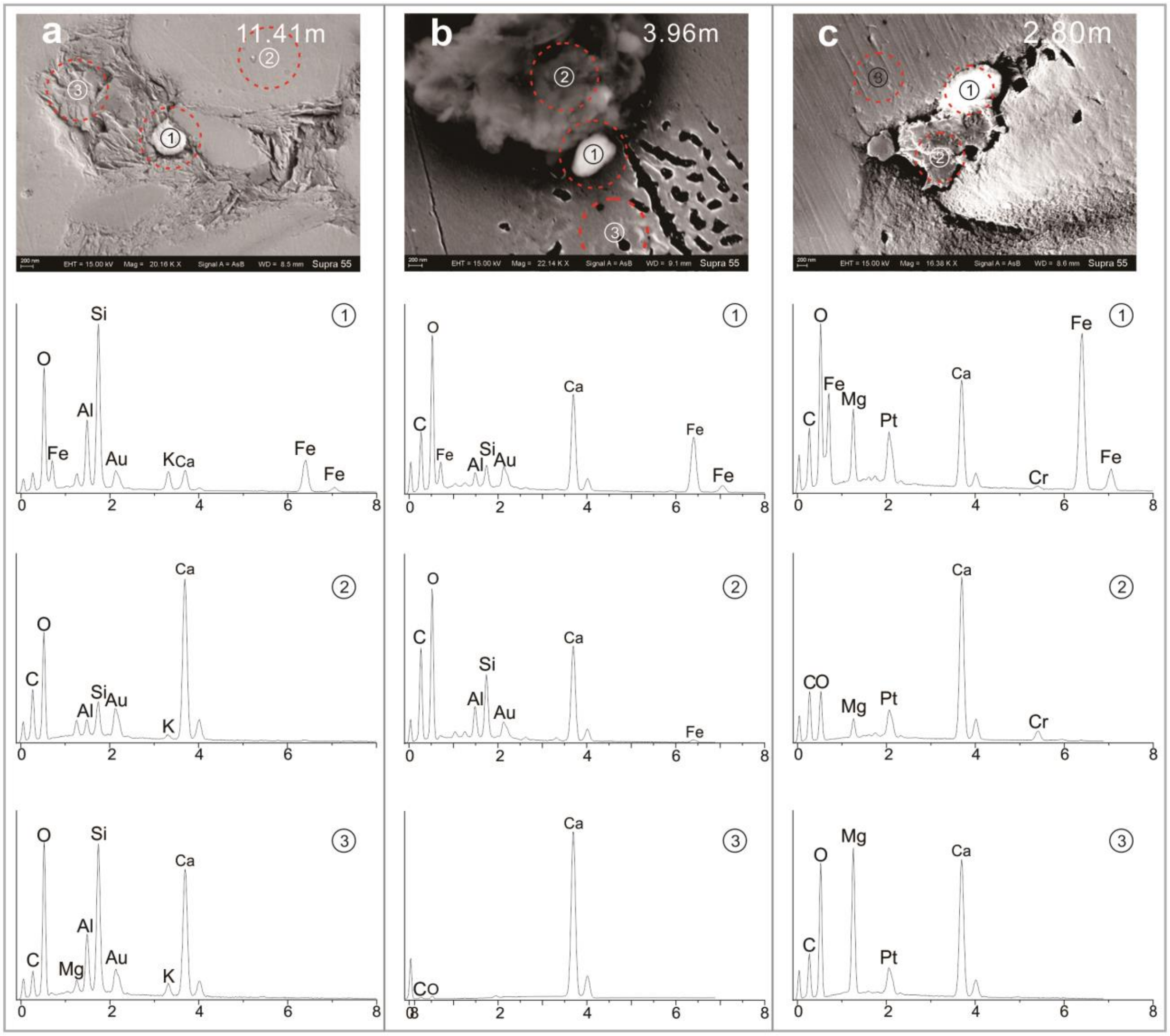

Figure S5 Part 2. SEM and EDS analyses of magnetite grains and their surrounding matrix, suggesting that Al and Cr in magnetite grains are likely from their surrounding matrix and represent substitutions. The diameter of a red dashed circle is 1.0 μm and the distance between EDS spots is greater than 1.0 μm to avoid contamination.

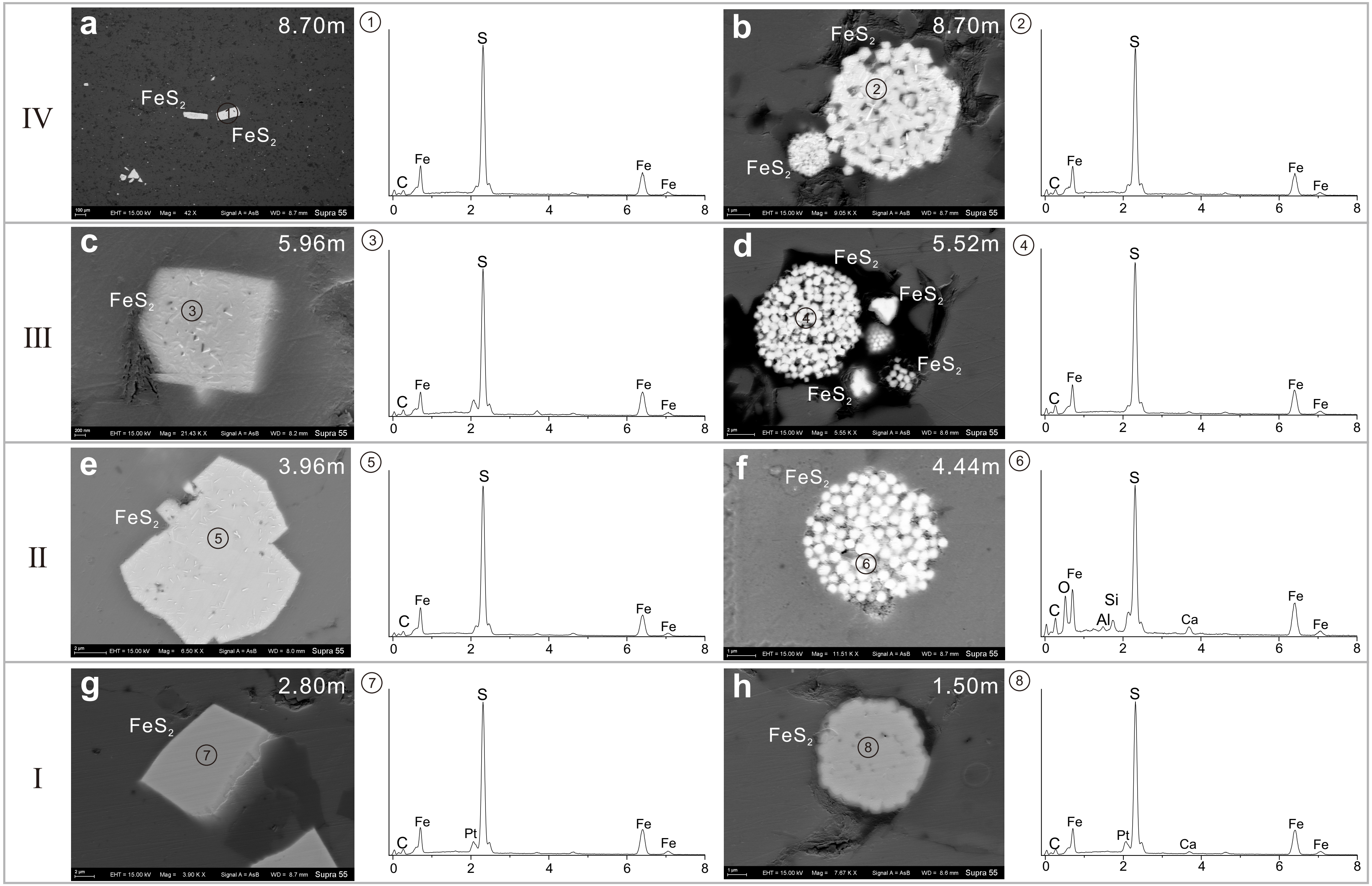

Figure S5 Part 3. SEM images of samples from the four intervals showing the occurrence of two types of pyrite grains, euhedral crystals and framboids of small, equal-sized crystal aggregates;

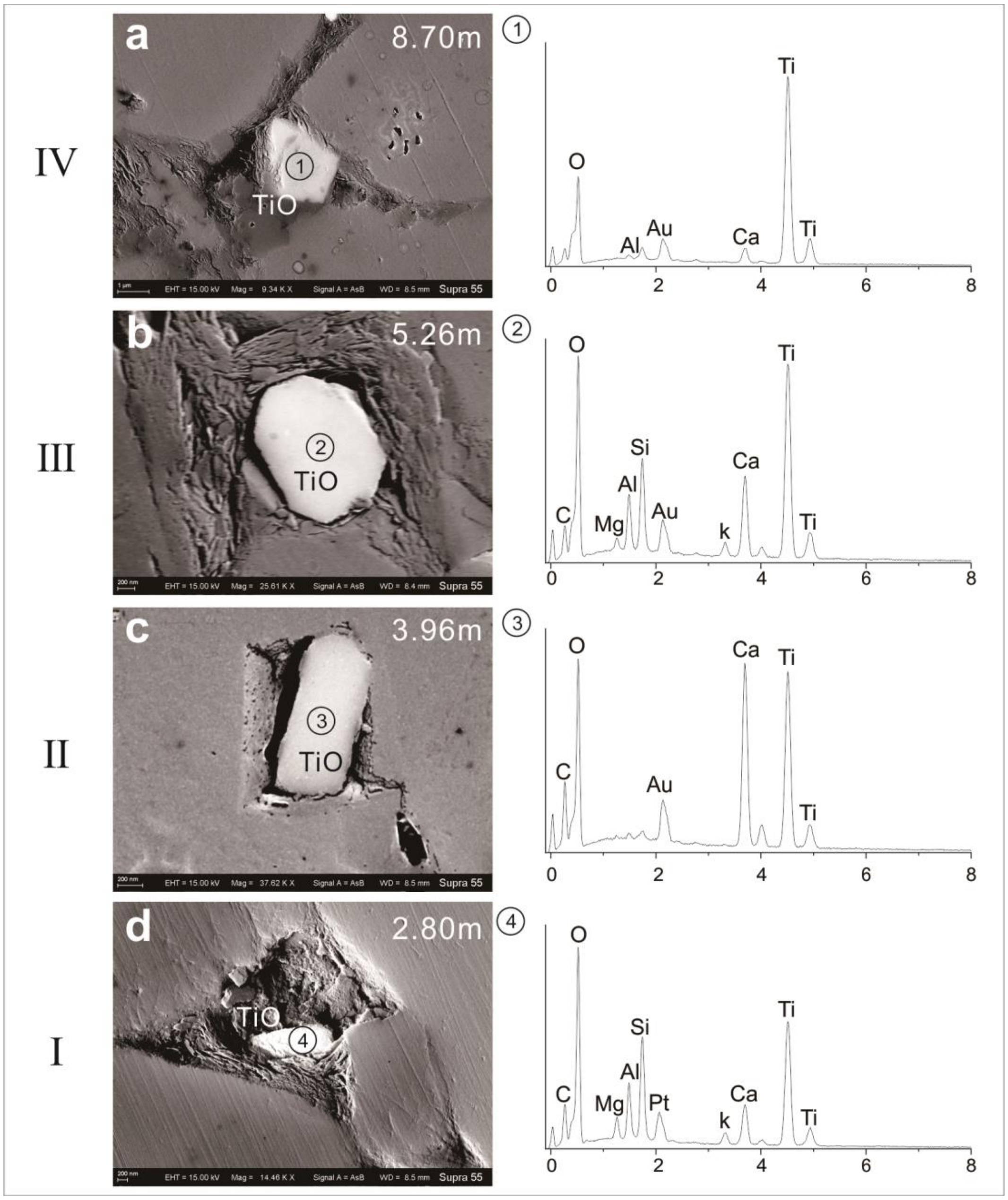

Figure S5 Part 4. SEM images showing the presence of detrital rutile (TiO_2) grains in the samples

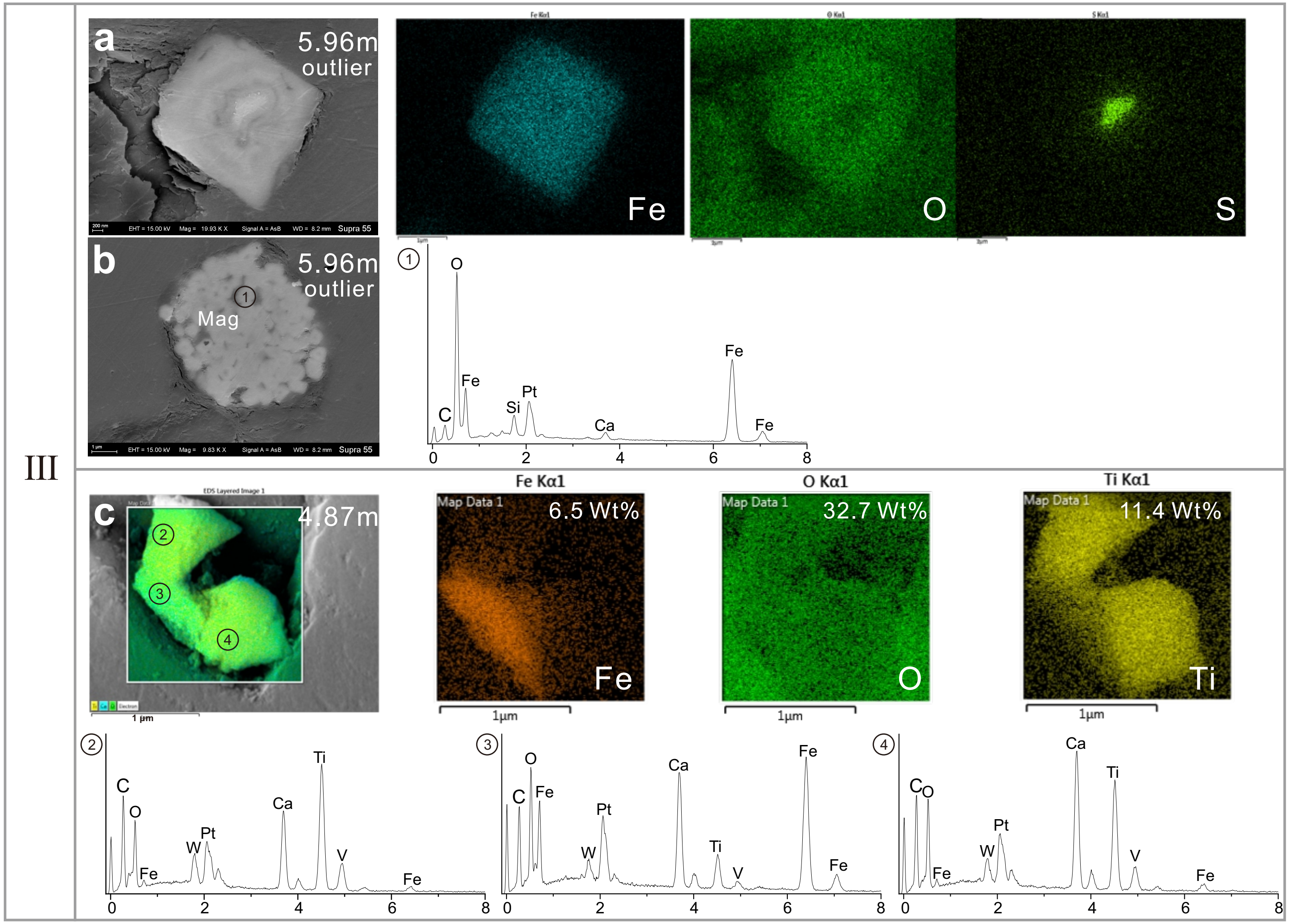

Figure S5 Part 5. SEM images of samples from Interval III showing the apparent co-occurrence of magnetite and sulfide (**a**), and transformation of pyrite to magnetite (**b**) in the sample at 5.96 m which shows an outlier direction, and the occurrence of titanomagnetite in the sample at 4.87 m (**c**).

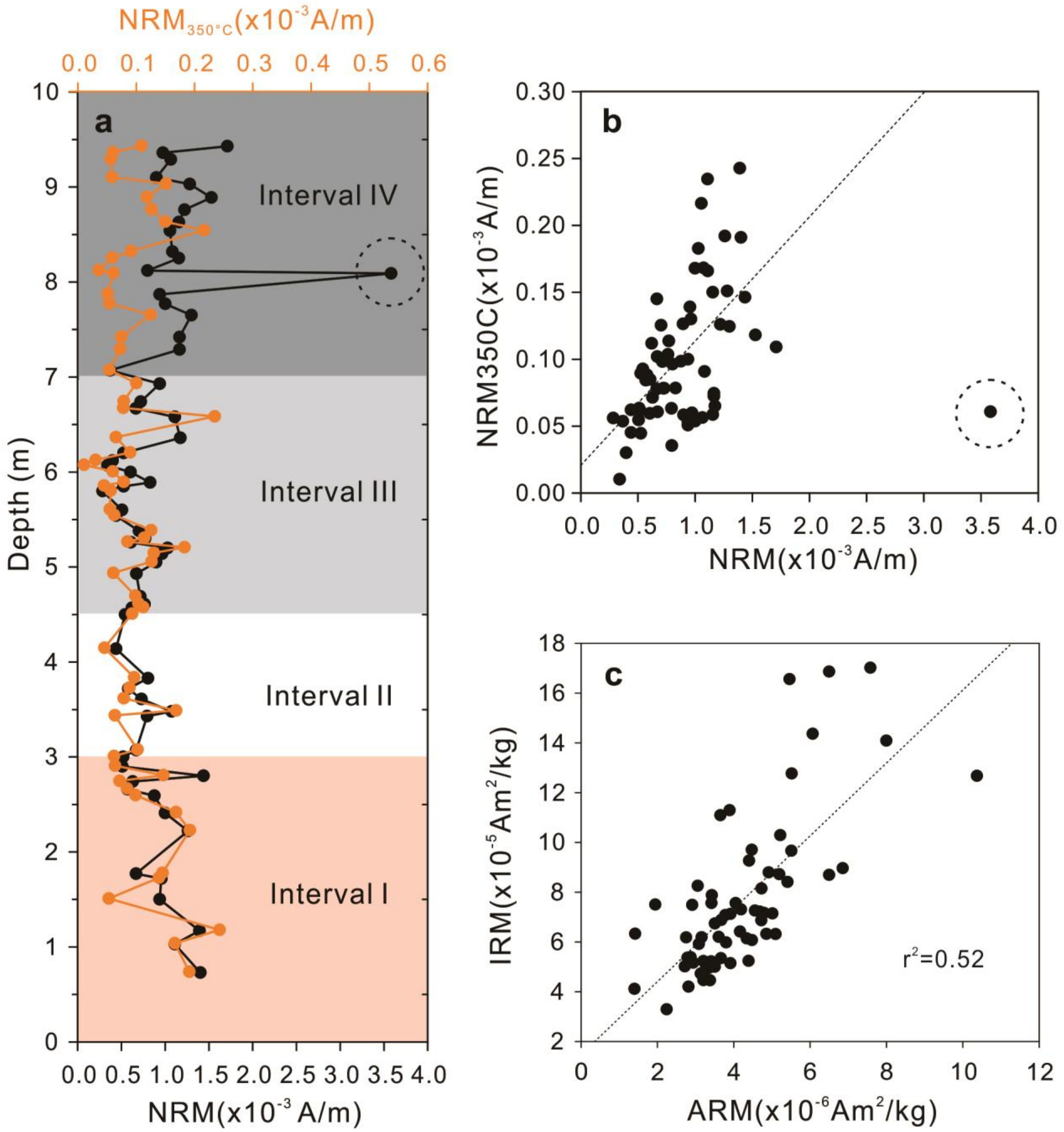

Figure S6. Changes of NRM and $NRM_{350°C}$, the start of HTC, of thermally demagnetized samples with depth showing that NRM is proportional to $NRM_{350°C}$ except at ~8.0 m (**a, b**). (**c**) The IRM and ARM data of rock magnetic samples from the ~9.5 m interval show a positive correlation. Therefore, the NRM/ARM and NRM/IRM shown in Fig. 2f, g can be used to detect anomalous intensity in the studied interval.

4.3 Paleomagnetic results

Natural remanent magnetizations (NRMs) of the samples range from 1.33×10^{-4} to 1.02×10^{-2} A/m (or 1.46×10^{-9} to 1.12×10^{-7} Am^2). Thermal demagnetization reveals predominantly a three-component magnetization (Fig. 1). The low-temperature component (LTC) is removed by ~200°C and the intermediate temperature component (ITC) is usually isolated by ~350°C. The remaining relatively high-temperature segment of the demagnetization trajectory decaying toward the origin is defined as the high temperature component (HTC) or characteristic remanent magnetization (ChRM). The magnetic intensities of most specimens drop to <2-3% of NRM intensity at ~470 or 480°C and about a dozen specimens show an intensity increase and a deviated trajectory from the origin at 520°C. So a temperature below 500°C is chosen as the end temperature step when fitting HTC directions. These features suggest that while hematite might be present in some samples, it is not a major contributor to the remanence.

The LTC, ITC, and HTC show similar directions to those reported in Jiao et al. (2018)[35]: the LTC documents a recent overprint and the ITC represents a Mesozoic overprint (Fig. S7). The HTC exhibits both normal and reversed polarities (Figs. 1, 3) (Table S1). A reversal test was performed for the ChRMs from the studied section excluding those from the polarity transition zone (i.e., interval III) and passes at 95% confidence level with class "B"[36] (Fig. 1e). Since the ChRMs are not perfectly circularly distributed (Fig. 1e), a Bingham statistical analysis was also performed, yielding a mean of Dec=36.2°, Inc=43.4°, α_{95min}=3.3°, α_{95max}=4.2°, which is similar to the means calculated using Fisher statistics. In addition, the paleomagnetic data from the polarity transition zone from ~4.5 m to 7.1 m (Interval III) exhibit demagnetization behavior similar to those of other Intervals, i.e., displaying three-component magnetization (Fig. S8). Moreover, the rock magnetic data across the polarity transition zone from ~4.5 m to 7.1 m (Interval III) show no dramatic changes that could result from secondary magnetic minerals. Together with a positive fold test for the paleomagnetic data from both the NW and SE limbs of the fold in this area (Fig. S1) from the previous study[35], these results further indicate that the studied interval preserves a record of the Cambrian geomagnetic field. In addition, the lithology of Intervals I to III do not suggest any major changes that might lead to differential compaction and different inclination shallowing. It is true

that even with a constant flattening factor inclinations could be affected differentially due to the functional relationship of inclination shallowing[37]. However, the dynamic range of the VGP shift supersedes any detailed application of a correction for inclination shallowing, and clearly inclination shallowing cannot cause reversed directions and other large changes in declination or the range of inclinations observed. It is also worth noting that this is a very high-resolution magnetostratigraphic study and in many cases, the sample spacing is only a few cm, almost close to or smaller than the size of a standard cylindrical paleomagnetic specimen. Variability in the data from a bed reflects differences in time and a highly erratic field (Table S1), especially during the hyper-reversal state of Interval I. For instance, at the nominal 3.0 m level where one sample shows a normal and the other shows a reversed polarity (Table S1) because one sample was drilled from an oriented block sample at 3.0 m and the other was a minicore drilled in the field at 3.0 m.

Limestone shows relatively weak magnetism and thermal alteration could occur during lab heating that would cause remanence increase, which presumably reflects the propensity of newly formed magnetic minerals to record stray internal and external fields, and great divergence of a demagnetization trajectory from the origin. About a dozen of specimens from Interval IV show this feature at above 500°C. These specimens were excluded for analyzing ChRMs except one at 9.21 m where demagnetization steps below 500°C were used to obtain the ChRM (Table S1). So, the ChRMs (Table S1) are not contributed to or affected by high-coercivity phases and by alteration during lab heating. In fact, thermal demagnetization is very effective and the intensity of most samples dropped below 2-3% of the initial NRM intensity by ~470 or 480°C. Similar features and reliable ChRMs were observed in the latest Permian limestone in South China as well[38].

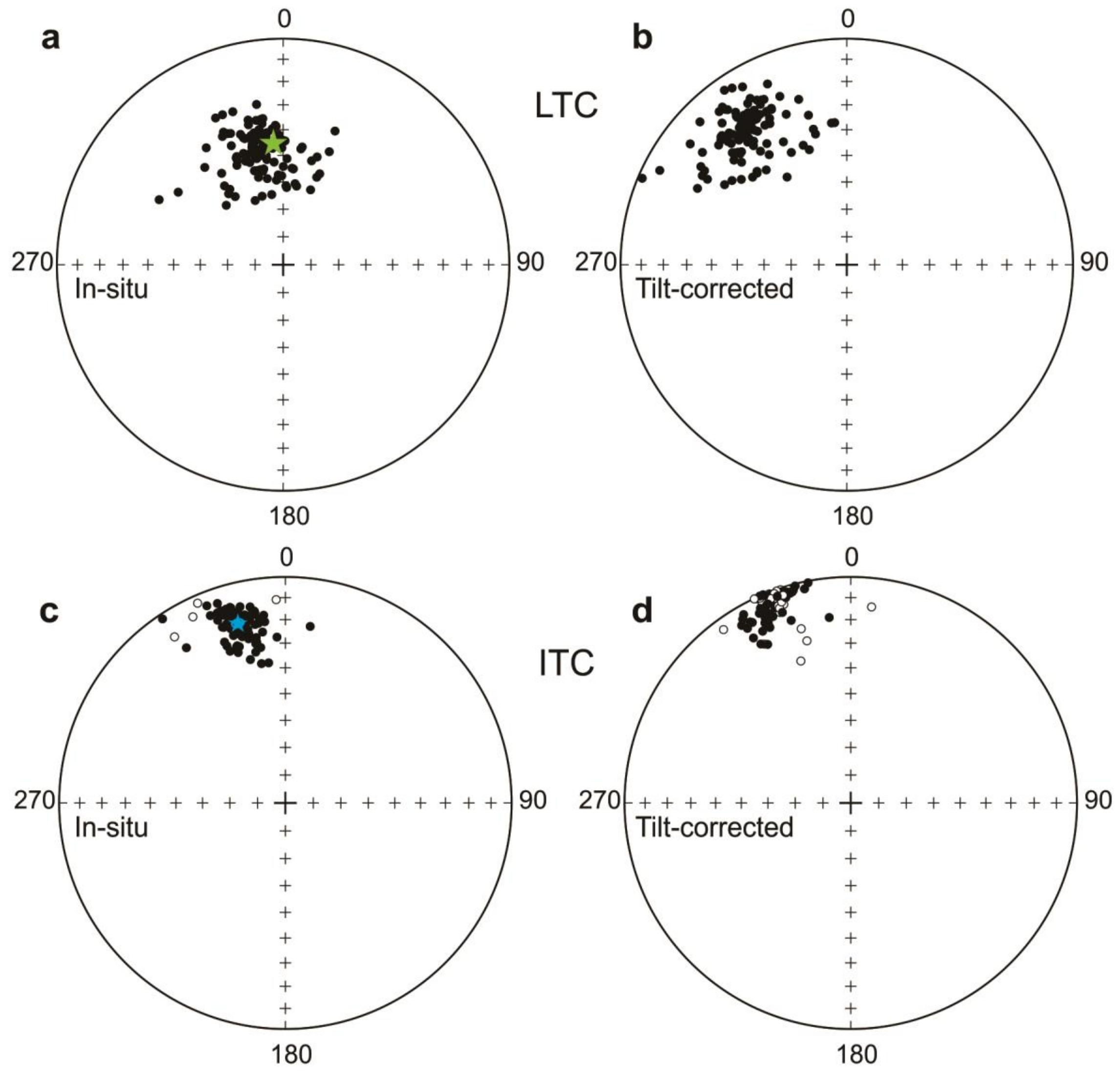

Figure S7. The equal-area stereographic projections of the LTCs and ITCs in geographic (**a, c**) and stratigraphic coordinates (**b, d**). The LTCs and ITCs are the same as those reported in Jiao et al (2018, ref.[35]), representing the recent overprint and the Mesozoic overprint, respectively.

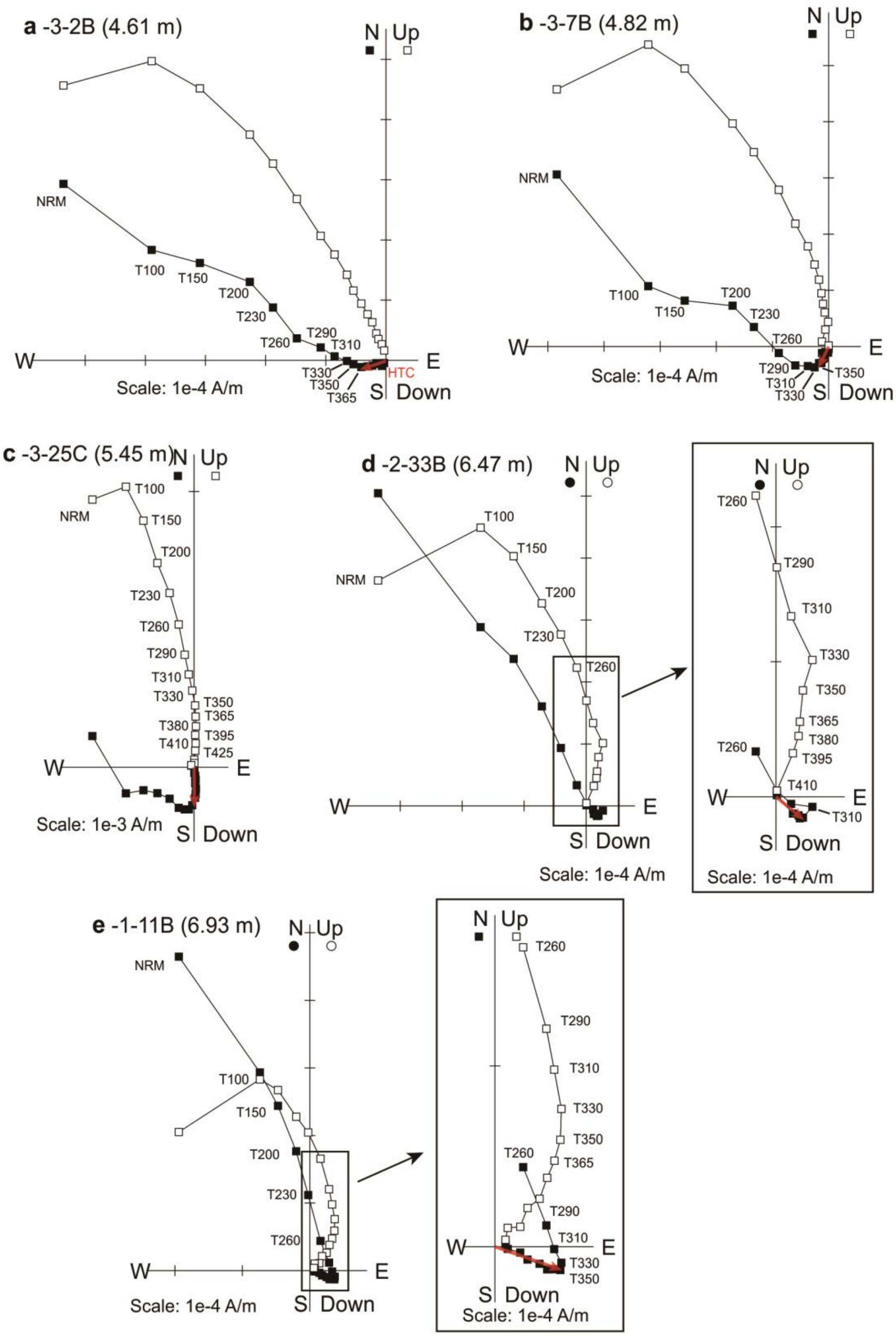

Figure S8. Representative demagnetization plots (stratigraphic coordinates) of specimens from Interval III.

Figure S9. Marine biodiversity, ocean-atmospheric O_2 level, and geomagnetic field evolution trends across the studied interval of late Cambrian. **a,** Increase in the Paleozoic genus and species diversity in Ordovician[39]. GOBE, the Great Ordovician Biodiversification Event. From Ref39. Reprinted with permission from AAAS. **b,** Estimates of Paleozoic atmospheric and oceanic oxygen level[40]. From Ref40. Reprinted with permission from AAAS. **c,** Dipole field strength of the geomagnetic field[2,8,41]. The paleointensity data of 200-500 Ma (Q>3) (triangles) are from ref[8,41], whereas other data are from ref[2]. The Ediacaran intensity value is a time-averaged paleomagnetic dipole moment[2]. **d,** Magnetic reversal frequency[42,43]. The dash, blue curves shown in late Devonian are based on ref[42].

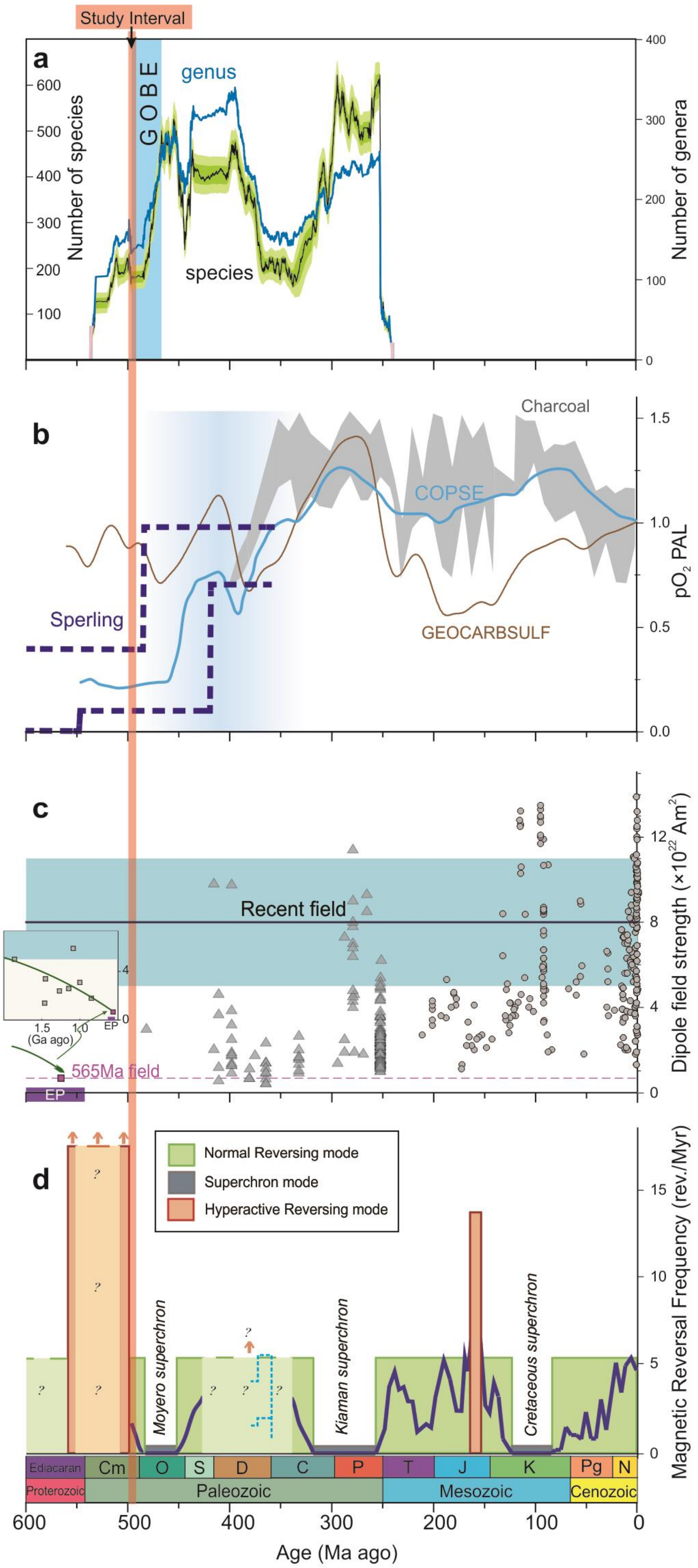

Supplementary References

1. Landeau, M., Aubert, J. & Olson, P. The signature of inner-core nucleation on the geodynamo. *Earth Planet. Sci. Lett.* **465**, 193–204 (2017).

2. Bono, R. K., Tarduno, J. A., Nimmo, F. & Cottrell, R. D. Young inner core inferred from Ediacaran ultra-low geomagnetic field intensity. *Nat. Geosci*. **12**, 143–147 (2019).

3. Zhou, T. *et al.* Early Cambrian renewal of the geodynamo and the origin of inner core structure. *Nat. Comm.* https://doi.org/10.1038/s41467-022-31677-7 (2022).

4. Dunlop, D. J. & Özdemir, Ö. Rock Magnetism, Fundamentals and Frontiers (Cambridge University Press, New York, NY, 1997).

5. Zhang, Y. *et al*. High geomagnetic field intensity recorded by anorthosite xenoliths requires a strongly powered late Mesoproterozoic geodynamo. *Proc. Natl. Acad. Sci.*, **119**, https://doi.org/10.1073/pnas.2202875119 (2022).

6. Macouin, M. *et al*. Low paleointensities recorded in 1 to 2.4 Ga Proterozoic dykes, Superior Province, Canada. *Earth Planet. Sci. Lett*. **213**, 79–95 (2003).

7. Smirnov, A. V. & Tarduno, J. A. Thermochemical remanent magnetization in Precambrian rocks: Are we sure the geomagnetic field was weak? *J. Geophys. Res.* **110**: B06103, doi: 10.1029/2004JB003445 (2005).

8. Hawkins, L. M. A. *et al*. Intensity of the Earth's magnetic field: Evidence for a Mid-Paleozoic dipole low. *Proc. Natl. Acad. Sc*i., **118** (2021).

9. Tarduno, J. A., Cottrell, R. D. & Smirnov, A. V. High geomagnetic intensity during the mid-Cretaceous from Thellier analyses of single plagioclase crystals. *Science* **291**, 1779-1783 (2001).

10. Tarduno, J. A. & Cottrell, R. D. Dipole strength and variation of the time-averaged reversing and non-reversing geodynamo based on Thellier analyses of single plagioclase crystals. *J. Geophys. Res.* 110: B11101, doi: 10.101029/2005JB003970 (2005).

11. Kulakov, E. V., Smirnov, A. V. & Diehl, J. F. Absolute geomagnetic paleointensity as recorded by ~1.09 Ga Lake Shore Traps (Keweenaw Peninsula, Michigan). *Studia Geophys. Geod.* **57**, 565-584 (2013).

12. Lloyd, S. J., Biggin, A. J., Paterson, G. A. & McCausland, P. J. A. Extremely weak early Cambrian dipole moment similar to Ediacaran: Evidence for long-term trends in geomagnetic field behaviour? *Earth Planet. Sci. Lett*. **595**, 117757 (2022).

13. Li, Z., Li, X., Zhou, H. & Kinny, P. D. Grenvillian continental collision in south China: New SHRIMP U-Pb zircon results and implications for the configuration of Rodinia. *Geology* **30**, 163–166 (2002).

14. Zhao, G. & Cawood, P. A. Precambrian geology of China. *Precambrian Res.* **222–223**, 13–54 (2012).

15. Shu, L., Wang, J. & Yao, J. Tectonic evolution of the eastern Jiangnan region, South China: New findings and implications on the assembly of the Rodinia supercontinent. *Precambrian Res*. **322**, 42–65 (2019).

16. Gilder, S. A., Keller, G. R., Luo, M. & Goodell, P. C. Eastern Asia and the western Pacific timing and spatial distribution of rifting in China. *Tectonophysics* **197**, 225–243 (1991).

17. Shu, L. *et al*. Neoproterozoic plate tectonic process and Phanerozoic geodynamic evolution of the South China Block. *Earth-Sci. Rev*. **216**, 103596 (2021).

18. Chen, X., Zhang, Y., Fan, J., Tang, L. & Sun, H. Onset of the Kwangsian Orogeny as evidenced by biofacies and lithofacies. *Sci. China: Earth Sci*. **55**, 1592–1600 (2012).

19. Peng, S. Tremadoc stratigraphy and trilobite faunas of northwestern Hunan. *Beringeria* **2**, 1−171 (1990).

20. Huang, R. *et al*. Ediacaran-Ordovician landscape of eastern South China: Constraints from sedimentary indices and detrital zircon U-Pb-Hf isotopes from the southeastern margin of the Yangtze Block. *Sediment. Geol.* **416**, 105865 (2021).

21. Yang, A., Zhu, M. & Zhang, J. Stratigraphic distribution and palaeogeographic control on the Early Cambrian Eodiscoids in Yangtze Platform. *J. Palaeogeogr.* **7**, 219-232 (2005).

22. Peng, S. *et al*. Global Standard Stratotype-Section and Point (GSSP) for the base of the Jiangshanian Stage (Cambrian: Furongian) at Duibian, Jiangshan, Zhejiang, Southeast China, *Episodes*, **35**, 462–

477 (2012b).
23. Peng, S., Babcock, L. E. & Cooper, R. A. In *Chapter 19 - the Cambrian Period* (ed. Gradstein, F.M.) 437–488 (Elsevier, 2012a).
24. BGMRZJ (Bureau of Geology and mineral Resources Zhejiang Province), Regional Geology of Zhejiang Province. Geology Publishing House, Beijing 5–575 (In Chinese with English Abstract) (1989).
25. Shu, L. An analysis of principal features of tectonic evolution in South China Block. *Geol. Bull. China* **31 (7)**, 1035–1053 (in Chinese with English abstract) (2012).
26. Peng, S. *et al.* Proposed GSSP for the base of Cambrian Stage 9, coinciding with the first appearance of *Agnostotes orientalis*, at Duibian, Zhejiang, China. *Sci. in China, Series D, Earth Sci.* **52**, 434–451 (2009).
27. Peng, S. C., Babcock, L. E. & Ahlberg, P. The Cambrian Period. *Geological Time Scale 2020*. **Chapter 19**, 565–629 (2020).
28. Zhao, Z. F. *et al*. Synchronizing rock clocks in the late Cambrian. *Nat. Comm.* https://doi.org/10.1038/s41467-022-29651-4 (2022).
29. Tauxe, L., Mullender, T. A. T. & Pick, T. Potbellies, wasp-waists, and superparamagnetism in magnetic hysteresis. *J. Geophys. Res*. **101**, 571–583 (1996).
30. Tarduno, J. A. & Myers, M. A primary magnetization fingerprint from the Cretaceous Laytonville Limestone: Further evidence for rapid oceanic plate velocities. *J. Geophys. Res*. **99**, 21691–21703 (1994).
31. Channell, J. E. T. & McCabe, C. Comparison of magnetic hysteresis parameters of unremagnetized and remagnetized limestones *J. Geophys. Res.* **99**, 4613-4623 (1994).
32. Day, R., Fuller, M. D., & Schmidt, V. A. Hysteresis properties of titanomagnetites: grain size and composition dependence. *Phys. Earth Planet. Inter*. **13**, 260–266 (1977).
33. Dunlop, D. J. Theory and application of the Day plot (Mrs/Ms versus Hcr/Hc) 1. Theoretical curves and tests using titanomagnetite data. *J. Geophys. Res*. **107**. (2002a)

34. Dunlop, D.J. Theory and application of the Day plot (Mrs/Ms versus Hcr/Hc) 2. Application to data for rocks, sediments, and soils. *J. Geophys. Res*. **107**. (2002b).

35. Jiao, W., Li, Y. & Yang, Z. Paleomagnetism of a well-dated marine succession in South China: A possible Late Cambrian true polar wander (TPW). *Phys. Earth Planet. Inter*. **277**, 38–54 (2018). https://doi.org/10.1016/j.pepi.2018.01.009

36. McFadden, P. L. & McElhinny, M. W. Classification of the reversal test in paleomagnetism. *Geophys. J. Int*., **103**, 725–729 (1990).

37. Tarduno, J.A. Absolute inclination values from deep sea sediments: a reexamination of the Cretaceous Pacific record. *Geophys. Res. Lett.* **17**, 101-104 (1990).

38. Zhang, M. *et al*. Magnetostratigraphy across the end-Permian mass extinction event from the Meishan sections, southeastern China. *Geology* **49**, 1289–1294, https://doi.org/10.1130/G49072.1 (2021).

39. Fan, J. *et al*. A high-resolution summary of Cambrian to Early Triassic marine invertebrate biodiversity. *Science* **367**, 272–277 (2020). https://doi.org/10.1126/science.aax4953

40. Lu, W. *et al*. Late inception of a resiliently oxygenated upper ocean. *Science* **361**, 174–177 (2018). https://doi.org/10.1126/science.aar5372

41. Hawkins, L. M. A. *et al*. An exceptionally weak Devonian geomagnetic field recorded by the Viluy Traps, Siberia. *Earth Planet. Sci. Lett*. **506**,134–145 (2019).

42. Hansma, J. *et al*. Late Devonian carbonate magnetostratigraphy from the Oscar and Horse Spring Ranges, Lennard Shelf, Canning basin, Western Australia. *Earth Planet. Sci. Lett*. **409**, 232–242 (2015).

43. Gallet, Y., Pavlov, V. & Korovnikov, I. Extreme geomagnetic reversal frequency during the Middle Cambrian as revealed by the magnetostratigraphy of the Khorbusuonka section (northeastern Siberia). *Earth Planet. Sci. Lett*. **528**. 115823 (2019). https://doi.org/10.1016/j.epsl.2019.115823

Supplementary

Table S1 Summary of paleomagnetic data from the studied section of the Huayansi Fm at the Jiangshanian GSSP in Zhejiang Province, China

GSSP-Height (m)	ID	CODE	STEP RANGE	Ds (°)	Is (°)	MAD (°)	VGP_Lat. (°)	VGP_long. (°)
26.5	2650-1	DirOPCA	T330-T450	14.3	37.9	5.2	75.0	235.6
26.5	2650-2	DirOPCA	T330-T430	10.2	32.3	4.8	75.4	256.7
26.5	2650-3	DirOPCA	T330-T390	11.0	35.9	1.9	76.6	247.8
26.5	2650-4	DirOPCA	T330-T430	1.3	42.5	6.6	85.7	282.8
22	J2200	DirOPCA	T380-T430	3.4	51.1	10.1	85.8	162.3
21.4	J2140	DirOPCA	T380-T430	54.5	57.0	5.1	44.2	182.7
21	2100-1	DirOPCA	T350-T450	7.7	18.4	6.3	69.3	276.6
21	2100-2	DirOPCA	T350-T450	16.3	21.7	6.4	66.8	254.3
21	2100-4	DirOPCA	T330-T430	7.6	17.6	7.9	69.0	277.3
20.5	J2050	DirOPCA	T350-T430	46.5	41.0	3.6	48.2	204.7
19.2	J1920	DirOPCA	T380-T430	77.8	47.1	5.1	23.0	187.9
18.5	J1850	DirOPCA	T380-T430	60.9	32.2	4.0	33.4	205.9
17.7	J1770	DirOPCA	T380-T430	3.6	30.3	3.8	77.1	283.0
17	1700-1	DirOPCA	T330-T450	4.6	32.7	8.6	78.2	276.6
17	1700-2	DirOPCA	T330-T410	22.7	45.5	6.5	70.5	208.8
17	1700-3	DirOPCA	T330-T410	18.2	24.3	7.9	66.7	248.4
17	1700-4	DirOPCA	T330-T450	17.6	24.9	2.8	67.3	248.8
16.9	J1690	DirOPCA	T350-T410	356.8	15.7	6.3	69.0	307.5
16.5	J1525	DirOPCA	T350-T430	60.0	45.7	6.5	37.6	195.1
16.25	J1500	DirOPCA	T350-T430	43.7	34.0	12.0	49.0	213.0
13.65	J1365	DirOPCA	T380-T430	58.3	42.4	5.1	38.2	198.8
13.4	J1340	DirOPCA	T380-T430	64.2	31.2	11.0	30.3	205.1
13.4	1340-1	DirOPCA	T350-T410	71.0	15.4	8.9	20.4	210.8
13.4	1340-2	DirOPCA	T350-T430	67.6	23.6	7.9	25.4	208.1
13.4	1340-3	DirOPCA	T350-T410	72.2	9.5	7.8	17.9	213.0
13.4	1340-4	DirOPCA	T350-T410	66.6	15.1	7.1	24.2	213.2
12.8	J1280	DirOPCA	T380-T430	47.4	63.9	3.1	49.4	170.9
12.35	J1235	DirOPCA	T380-T450	348.2	70.1	7.9	63.3	103.1
11.75	J1175	DirOPCA	T380-T430	46.4	46.6	6.3	49.4	198.3
11.2	J1120	DirOPCA	T380-T450	74.1	52.7	8.6	27.7	183.9
11.44	2-18B	DirOPCA	T350-T425	22.7	36.7	4.8	67.8	225.6
11.41	2-17B	DirOPCA	T330-T410	358.8	34.7	2.2	80.2	305.3
11.33	2-16A	DirOPCA	T380-T440	52.8	41.5	3.6	42.8	201.7
11.23	2-14B	DirOPCA	T380-T440	28.2	49.7	2.9	65.5	197.6
11.14	2-12	DirOPCA	T380-T440	15.3	33.4	2.1	72.4	244.4
10.9	2-7B	DirOPCA	T310-T440	5.7	30.7	6.5	76.7	274.2
10.9	2-7A	DirOPCA	T350-T425	17.2	29.9	6.0	69.7	243.8
10.84	2-6B	DirOPCA	T365-T440	8.6	25.9	4.2	72.9	269.0
10.84	2-6A	DirOPCA	T350-T425	46.4	38.6	5.4	47.7	207.2
10.75	2-4	DirOPCA	T380-T455	2.5	52.2	3.8	85.5	146.2
10.67	2-3B	DirOPCA	T365-T455	32.1	54.2	8.8	62.2	188.0
10.67	2-3A	DirOPCA	T350-T440	25.1	42.5	3.5	67.2	213.3
10.56	2-1B	DirOPCA	T380-T455	52.7	16.7	6.7	36.6	220.0
10.56	2-1A	DirOPCA	T380-T425	58.0	49.8	5.4	40.2	191.4
10.49	1-18A	DirOPCA	T365-T455	349.7	54.7	8.9	79.2	67.6
10.33	1-16B	DirOPCA	T380-T470	354.8	27.1	4.4	74.8	318.1
10.24	1-14A	DirOPCA	T350-T455	5.8	52.6	4.5	83.4	165.7
10.21	1-13B	DirOPCA	T330-T410	46.7	39.5	3.4	47.7	206.2
10.18	1-12C	DirOPCA	T365-T455	12.3	71.6	3.2	61.1	132.7
10.14	1-11B	DirOPCA	T350-T425	24.6	59.1	6.1	67.0	173.4
10.11	1-10B	DirOPCA	T330-T410	47.9	40.2	3.6	46.8	205.0
9.94	1-7B	DirOPCA	T365-T440	359.0	32.1	5.2	78.6	303.4
9.91	1-6B	DirOPCA	T330-T410	30.9	39.4	3.7	61.5	214.5
9.82	1-4B	DirOPCA	T350-T425	54.1	28.2	4.6	38.4	212.0
9.48	0-27B	DirOPCA	T365-T425	42.3	38.0	5.6	51.2	209.8
9.43	0-26A	DirOPCA	T380-T455	8.5	71.5	3.9	61.9	128.7
9.36	0-24C	DirOPCA	T350-T440	36.3	42.7	5.4	57.5	207.0
9.33	0-23C	DirOPCA	T365-T440	28.0	45.3	6.8	65.2	206.5
9.29	0-22C	DirOPCA	T365-T455	65.3	39.1	6.7	31.4	198.9
9.29	0-22A	DirOPCA	T380-T425	74.4	39.8	3.8	23.8	194.9
9.28	0-21B	DirOPCA	T365-T440	58.6	40.9	3.2	37.6	200.0
9.21	0-19B	DirOPCA	T330-T425	349.6	51.1	4.3	80.5	49.6
9.13	0-17	DirOPCA	T350-T440	350.3	60.8	3.5	74.8	90.0
9.1	0-16C	DirOPCA	T350-T425	76.9	37.9	7.2	21.1	195.2
9.07	0-15A	DirOPCA	T350-T425	36.5	67.1	2.5	55.3	161.1
8.92	0-11A	DirOPCA	T350-T440	66.7	58.6	4.8	35.0	178.7
8.89	0-10B	DirOPCA	T365-T470	37.8	75.9	5.2	47.8	142.8
8.81	0-8B	DirOPCA	T330-T425	31.0	38.3	4.1	61.1	216.0
8.7	0-6A	DirOPCA	T365-T440	44.7	57.0	4.3	51.9	183.3
8.7	0-5A	DirOPCA	T330-T425	48.2	32.9	5.5	44.7	211.5
8.66	0-4B	DirOPCA	T365-T455	40.4	39.2	3.5	53.1	209.4
8.63	0-3B	DirOPCA	T330-T470	23.9	28.4	6.0	64.0	235.3
8.54	0-1C	DirOPCA	T330-T425	343.9	27.0	3.1	69.2	347.8
8.32	-0-18C	DirOPCA	T350-T440	22.9	35.2	4.4	67.1	227.7
8.29	-0-17B	DirOPCA	T330-T455	42.6	35.6	3.4	50.3	212.1
8.25	-0-16C	DirOPCA	T365-T440	32.4	26.4	5.1	56.5	228.1
8.15	-0-14B	DirOPCA	T350-T455	42.1	37.4	4.8	51.2	210.5

8.12	-0-13B	DirOPCA	T380-T425	61.0	29.9	4.8	32.8	207.4
8.09	-0-12	DirOPCA	T380-T440	11.4	32.9	2.1	94.9	252.3
8.03	-0-11C	DirOPCA	T380-T440	65.9	38.7	3.8	30.8	199.0
7.96	-0-10C	DirOPCA	T380-T425	67.3	39.9	7.0	29.9	197.5
7.94	-0-9B	DirOPCA	T350-T440	52.2	48.9	5.6	44.9	193.9
7.87	-0-7C	DirOPCA	T380-T440	69.6	37.3	5.1	27.2	198.6
7.83	-0-6B	DirOPCA	T350-T455	23.4	46.0	5.5	69.3	206.9
7.77	-0-5C	DirOPCA	T380-T455	66.0	33.5	6.9	29.3	202.7
7.72	-0-4B	DirOPCA	T350-T425	13.8	37.3	3.5	75.2	237.9
7.65	-0-3A	DirOPCA	T330-T425	32.8	36.6	4.3	59.1	217.0
7.5	-0-1B	DirOPCA	T365-T425	67.3	46.3	3.5	31.5	192.1
7.42	-1-19A	DirOPCA	T365-T440	42.4	44.5	5.1	52.5	202.3
7.37	-1-18B	DirOPCA	T330-T455	36.8	75.4	3.2	48.7	143.4
7.29	-1-17	DirOPCA	T350-T455	49.9	37.2	4.1	44.3	206.9
7.26	-1-16B	DirOPCA	T350-T455	30.9	65.5	3.4	59.6	161.7
7.07	-1-15B	DirOPCA	T330-T440	108.1	-46.5	7.0	-27.7	226.8
7.04	-1-14B	DirOPCA	T365-T440	112.4	-45.8	4.7	-31.1	224.7
6.93	-1-11B	DirOPCA	T365-T455	110.9	-50.2	4.8	-31.1	229.4
6.93	-1-10	DirOPCA	T395-T470	122.4	-39.8	3.5	-38.2	215.8
6.88	-1-9A	DirOPCA	T350-T440	99.5	-56.7	8.5	-24.0	239.4
6.85	-1-8B	DirOPCA	T350-T440	104.3	-55.1	7.4	-27.2	236.3
6.77	-1-7	DirOPCA	T395-T440	116.8	-40.4	3.0	-33.5	218.5
6.74	-1-6B	DirOPCA	T330-T440	111.2	-62.5	9.3	-34.2	244.2
6.67	-1-4A	DirOPCA	T365-T440	105.0	-75.5	3.6	-32.1	267.0
6.61	-1-3A	DirOPCA	T365-T410	139.0	-62.4	2.9	-54.2	244.5
6.58	-1-2B	DirOPCA	T350-T440	151.8	-79.3	1.9	-46.4	284.6
6.52	-1-1B	DirOPCA	T365-T425	150.9	-50.9	3.2	-64.8	222.3
6.47	-2-33B	DirOPCA	T365-T410	131.7	-64.9	2.8	-48.6	248.1
6.41	-2-32A	DirOPCA	T365-T440	141.8	-64.7	7.6	-55.4	250.2
6.36	-2-31	DirOPCA	T350-T410	202.5	-59.1	3.6	-68.5	351.7
6.27	-2-30B	DirOPCA	T365-T440	142.2	-75.4	3.5	-48.3	273.4
6.12	-2-25	DirOPCA	T350-T425	92.4	-9.6	7.7	-4.4	211.7
6.11	-2-26	DirOPCA	T350-T425	46.2	0.2	5.6	37.4	233.3
6.08	-2-24	DirOPCA	T365-T440	105.0	-38.2	5.7	-22.8	221.4
6.07	-2-23	DirOPCA	T350-T395	89.9	16.4	7.6	4.1	201.3
5.96	-2-18A	DirOPCA	T330-T395	29.6	-48.8	13.2	25.0	270.4
5.91	-2-17	DirOPCA	T365-T410	120.0	-20.5	7.4	-31.3	203.5
5.88	-2-15	DirOPCA	T365-T425	120.5	-44.9	5.5	-37.8	221.2
5.88	-2-14A	DirOPCA	T330-T395	188.1	-73.0	7.8	-59.7	307.0
5.83	-2-12	DirOPCA	T330-T425	344.9	-54.7	5.4	24.2	313.1
5.81	-2-11	DirOPCA	T365-T425	139.8	-45.3	5.2	-54.6	215.2
5.78	-2-9B	DirOPCA	T380-T455	110.2	-48.1	3.9	-29.9	227.5
5.65	-2-6B	DirOPCA	T365-T440	119.8	-57.2	4.0	-39.7	235.5
5.65	-2-6A	DirOPCA	T330-T395	106.4	-63.5	4.3	-31.1	246.3
5.65	-2-5	DirOPCA	T350-T425	157.4	-57.6	4.8	-69.0	241.2
5.55	-2-3B	DirOPCA	T365-T455	139.6	-59.1	4.1	-55.2	238.0
5.54	-2-2	DirOPCA	T330-T395	214.5	-42.8	3.5	-59.1	27.7
5.52	-2-1A	DirOPCA	T380-T455	140.6	-53.3	5.3	-56.2	227.4
5.45	-3-25C	DirOPCA	T350-T455	176.5	-62.8	2.0	-74.3	289.3
5.38	-3-24B	DirOPCA	T365-T470	144.5	-58.8	2.4	-59.0	238.3
5.35	-3-23B	DirOPCA	T380-T470	150.2	-60.5	3.4	-62.7	244.2
5.3	-3-22B	DirOPCA	T395-T470	143.8	-57.7	3.2	-58.6	235.9
5.3	-3-21B	DirOPCA	T380-T470	153.2	-56.8	2.3	-66.1	236.6
5.26	-3-20B	DirOPCA	T380-T470	138.9	-63.5	3.8	-53.8	246.7
5.2	-3-17B	DirOPCA	T350-T440	163.3	-69.3	2.3	-62.9	276.2
5.2	-3-16B	DirOPCA	T380-T455	162.7	-65.3	3.6	-67.1	267.5
5.15	-3-15B	DirOPCA	T380-T455	161.6	-62.1	2.6	-69.3	258.2
5.14	-3-14	DirOPCA	T365-T440	171.6	-65.0	2.7	-70.7	281.1
5.09	-3-13B	DirOPCA	T330-T425	174.2	-69.1	3.4	-65.8	290.0
5.05	-3-12B	DirOPCA	T380-T440	172.3	-62.6	3.2	-73.6	278.6
4.96	-3-11	DirOPCA	T330-T410	204.5	-69.3	1.7	-59.9	328.5
4.93	-3-10B	DirOPCA	T365-T425	178.6	-75.3	3.6	-56.5	297.4
4.87	-3-9B	DirOPCA	T350-T440	180.8	-66.7	2.5	-69.5	301.0
4.85	-3-8B	DirOPCA	T350-T440	181.4	-69.0	3.5	-66.3	300.7
4.82	-3-7B	DirOPCA	T365-T455	202.5	-71.4	5.5	-58.4	322.7
4.77	-3-6B	DirOPCA	T380-T455	207.1	-66.2	2.2	-61.3	337.5
4.69	-3-5B	DirOPCA	T380-T455	210.0	-75.9	3.5	-50.5	319.2
4.68	-3-4	DirOPCA	T350-T440	211.6	-61.7	2.6	-61.0	351.0
4.65	-3-3	DirOPCA	T330-T440	242.2	-73.2	4.0	-38.6	334.4
4.61	-3-2B	DirOPCA	T365-T455	251.1	-67.5	3.3	-33.5	345.0
4.57	-3-1B	DirOPCA	T350-T470	285.4	-59.0	2.9	-7.4	347.0
4.5	-4-25	DirOPCA	T365-T440	213.5	-76.6	3.5	-48.5	319.6
4.44	-4-24B	DirOPCA	T380-T440	246.4	-23.4	2.8	-26.4	28.8
4.18	-4-22	DirOPCA	T350-T440	282.0	-62.7	5.6	-11.8	344.5
4.14	-4-21	DirOPCA	T365-T440	164.8	-80.4	6.0	-46.7	291.6
3.93*	-4-16B	DirOPCA	T365-T425	216.0	-70.4	5.5	-53.5	333.6
3.93*	-4-16A	DirOPCA	T350-T410	262.4	-24.7	2.2	-12.8	20.7
3.86	-4-14B	DirOPCA	T365-T455	208.3	-39.3	5.5	-63.7	36.5
3.83	-4-13B	DirOPCA	T380-T455	181.4	-54.4	3.2	-83.8	309.2
3.83	-4-13A	DirOPCA	T380-T470	203.4	-52.1	5.4	-69.6	11.7
3.78	-4-12B	DirOPCA	T380-T455	189.7	-51.7	3.5	-80.9	3.2
3.75	-4-11	DirOPCA	T365-T470	216.7	-40.7	4.5	-56.7	29.5

3.72	-4-10B	DirOPCA	T365-T455	197.4	-54.7	3.0	-73.9	0.6
3.69	-4-9B	DirOPCA	T395-T470	185.8	-54.4	3.4	-81.0	342.2
3.67	-4-7	DirOPCA	T365-T440	224.3	-26.8	2.7	-46.6	38.6
3.61	-4-6	DirOPCA	T365-T440	205.5	-41.8	3.9	-66.7	34.3
3.58	-4-5	DirOPCA	T330-T395	217.3	-30.9	3.1	-53.7	39.9
3.48	-4-4	DirOPCA	T310-T440	232.3	-40.0	7.5	-42.9	23.3
3.48*	-4-3	DirOPCA	T330-T425	233.7	-45.8	3.1	-43.0	17.0
3.43	-4-2	DirOPCA	T380-T440	219.2	-47.1	4.6	-55.7	19.8
3.43*	-4-1	DirOPCA	T330-T455	237.4	-45.3	2.2	-39.7	16.3
3.07	DB307	DirOPCA	T350-T440	162.9	-57.3	9.9	-73.1	245.7
3	DB300	DirOPCA	T350-T425	148.0	-55.8	9.7	-62.1	232.6
3	200-3	DirOPCA	T370-T450	49.8	45.3	12.9	46.2	198.8
2.9	DB290	DirOPCA	T310-T440	203.7	-57.7	7.6	-68.2	356.5
2.8	J280	DirOPCA	T380-T450	247.8	-27.4	3.2	-26.2	25.8
2.74	DB274	DirOPCA	T330-T425	179.5	-46.5	7.4	-88.9	142.2
2.66	DB266	DirOPCA	T350-T470	181.3	-31.0	13.7	-77.9	112.7
2.59	DB259	DirOPCA	T330-T410	68.6	-6.7	8.2	16.9	222.3
2.41	DB241	DirOPCA	T330-T440	206.4	-44.5	4.8	-66.5	28.7
2.4	140-1	DirOPCA	T330-T410	41.1	37.4	11.0	52.1	211.0
2.4	140-3	DirOPCA	T350-T450	16.0	28.9	8.0	70.1	247.3
2.4	140-4	DirOPCA	T350-T450	29.5	37.6	12.3	62.2	218.0
2.22	DB222	DirOPCA	T290-T425	212.5	-2.9	4.2	-48.7	64.2
1.77	DB177	DirOPCA	T290-T455	226.2	-32.1	5.7	-46.3	33.2
1.72	DB172	DirOPCA	T330-T425	209.7	-9.1	6.3	-52.9	63.7
1.5	50-4	DirOPCA	T350-T410	79.6	45.5	9.3	21.1	188.6
1.5	J150	DirOPCA	T350-T470	330.6	-20.7	3.4	41.4	338.6
1.17	J117	DirOPCA	T350-T450	224.9	-19.7	4.1	-44.1	43.2
1.03	DB103	DirOPCA	T310-T440	228.9	-21.0	5.2	-41.0	39.7
0.78	DB78	DirOPCA	T310-T470	330.5	-21.1	3.5	41.1	338.5
0.73	DB73	DirOPCA	T310-T440	243.4	-22.8	5.6	-28.9	30.6
0.2	-80-1	DirOPCA	T350-T450	78.8	47.3	12.3	22.3	187.4
0.2	-80-3	DirOPCA	T370-T450	75.7	45.1	7.9	24.2	190.3
0.2	-80-4	DirOPCA	T370-T450	71.0	54.5	11.0	30.7	182.8

* samples that yield reversed polarity in Jiao et al. (2018); MAD, maximum angular deviation;
VGP_lat., latitude of virtual geomagnetic pole; VGP_long., longitude of virtual geomagnetic pole

Interval IV: normal polarity
Interval III: polarity transition
Interval II: reversed polarity
Interval I: hyper-reversal polarities

Supplementary

Table S2 Rock magnetic data of the 0-9.5 m interval of the studied section at the Jiangshanian GSSP

Height (m)	NRM(x10^{-7}Am^2/kg)	ARM(x10^{-6}Am^2/kg)	IRM(x10^{-5}Am^2/kg)	S-ratio	ARM/IRM(x10^{-2})	NRM/ARM(x10^{-1})	NRM/IRM(x10^{-2})
9.43	2.89	4.34	6.13	0.83	7.08	0.67	0.47
9.36	20.53	3.89	11.29	0.89	3.45	5.27	1.82
9.29	4.71	4.73	8.14	0.66	5.80	1.00	0.58
9.10	4.44	10.37	12.67	0.84	8.18	0.43	0.35
9.03	11.92	5.22	10.29	0.82	5.07	2.28	1.16
8.89	27.83	7.58	17.02	0.88	4.45	3.67	1.64
8.76	4.52	5.41	8.41	0.77	6.43	0.84	0.54
8.63	3.06	5.51	9.66	0.42	5.70	0.55	0.32
8.54	3.16	3.78	7.07	0.28	5.34	0.84	0.45
8.32	14.04	8.00	14.09	0.77	5.68	1.75	1.00
8.25	2.74	5.01	7.14	0.89	7.02	0.55	0.38
8.12	5.36	3.61	6.20	0.80	5.82	1.49	0.86
8.09	5.28	6.50	16.86	0.17	3.85	0.81	0.31
7.87	1.34	4.68	7.21	0.84	6.49	0.29	0.19
7.77	19.74	5.46	16.56	0.85	3.30	3.61	1.19
7.65	5.23	5.52	12.76	0.44	4.33	0.95	0.41
7.42	3.40	3.21	4.46	0.76	7.19	1.06	0.76
7.29	3.09	4.91	8.79	0.62	5.59	0.63	0.35
7.07	1.03	2.25	3.29	0.88	6.83	0.46	0.31
6.93	4.80	6.07	14.37	0.57	4.22	0.79	0.33
6.74	3.20	4.05	7.56	0.87	5.36	0.79	0.42
6.67	2.86	3.41	5.21	0.88	6.54	0.84	0.55
6.58	3.82	3.51	6.74	0.73	5.20	1.09	0.57
6.36	5.95	5.19	8.72	0.59	5.96	1.15	0.68
6.20	3.05	4.72	6.87	0.89	6.88	0.65	0.44
6.12	3.54	3.21	5.23	0.77	6.13	1.10	0.68
6.07	15.56	4.47	9.70	0.88	4.61	3.48	1.60
6.00	3.43	4.17	6.42	0.86	6.49	0.82	0.53
5.89	1.59	2.87	5.38	0.87	5.34	0.55	0.30
5.85	1.99	2.79	5.37	0.89	5.20	0.71	0.37
5.80	6.11	3.42	7.56	0.82	4.52	1.79	0.81
5.60	2.40	3.13	4.72	0.85	6.64	0.77	0.51
5.54	3.70	2.91	7.48	0.76	3.89	1.27	0.49
5.38	4.47	3.06	8.25	0.81	3.71	1.46	0.54
5.30	1.94	4.56	7.25	0.80	6.28	0.43	0.27
5.26	3.70	3.16	6.18	0.70	5.11	1.17	0.60
5.20	3.86	4.79	7.16	0.72	6.70	0.80	0.54
5.14	9.64	3.65	11.09	0.90	3.29	2.64	0.87
5.05	4.49	6.85	8.96	0.89	7.65	0.66	0.50
4.93	10.88	3.42	7.88	0.88	4.35	3.18	1.38
4.69	5.76	3.66	5.34	0.88	6.86	1.57	1.08
4.61	2.39	2.72	5.01	0.85	5.42	0.88	0.48
4.57	5.08	3.26	4.90	0.82	6.66	1.56	1.04
4.50	4.47	1.40	4.11	0.93	3.40	3.20	1.09
4.14	15.16	3.09	5.92	0.88	5.22	4.91	2.56
3.83	3.58	4.19	7.30	0.88	5.74	0.85	0.49
3.72	3.26	3.92	5.14	0.85	7.61	0.83	0.63
3.61	6.83	3.91	7.12	0.91	5.50	1.74	0.96
3.48	7.69	4.40	9.26	0.92	4.75	1.75	0.83
3.43	2.57	1.42	6.33	0.84	2.24	1.82	0.41
3.07	0.49	2.94	5.16	0.81	5.69	0.17	0.10
3.00	2.88	3.80	5.98	0.79	6.36	0.76	0.48
2.90	0.81	2.82	4.20	0.80	6.71	0.29	0.19
2.80	2.67	3.38	4.46	0.77	7.58	0.79	0.60
2.74	3.19	2.75	6.18	0.84	4.46	1.16	0.52
2.66	0.81	3.51	5.00	0.81	7.02	0.23	0.16
2.59	3.67	5.09	6.31	0.87	8.07	0.72	0.58
2.41	4.30	4.48	6.07	0.62	7.37	0.96	0.71
2.22	2.51	4.86	6.32	0.84	7.69	0.52	0.40
1.77	6.61	3.67	6.88	0.93	5.34	1.80	0.96
1.72	1.87	1.95	7.50	0.90	2.60	0.96	0.25
1.50	3.91	4.39	5.24	0.85	8.38	0.89	0.75
1.17	2.03	6.50	8.69	0.85	7.47	0.31	0.23
1.03	0.30	3.35	4.96	0.83	6.76	0.09	0.06
0.73	3.56	3.43	5.00	0.87	6.85	1.04	0.71
